# A Theoretical and Practical Treatise on Midwifery, Including the Diseases of Pregnancy and Parturition

**Published:** 1850-08

**Authors:** 


*A Theoretical and Practical Treatise on Midwifery, including the diseases of pregnancy and parturition.* By P. Cazeaux, adjunct Professor in the Faculty of Medicine in Paris, etc., etc. Adopted by the Royal Council of Public Instruction. Translated from the second French edition, with occasional notes, and a copious index. By Robert P. Thomas, M. D., etc. With one hundred and seventeen illustrations. Philadelphia: Lindsay & Blackiston. 1850. 8 vo. pp. 765.

The treatise on midwifery by Cazeaux has acquired a high reputation, as evinced by its adoption by the Royal Council of Public Instruction, and by the demand for a second edition from which this translation has been made. The translator informs us in his prefatory remarks, that the new and highly interesting physiological developements respecting the changes that take place in the ovary and ovulum before and after fecundation, are so described and illustrated in this work, as to be intelligible to every reader. The arrangement and description of the mechanism of labor, peculiar to Cazeaux, he thinks is calculated to simplify the subject. The practical principles inculcated are based on an extensive hospital practice. We do not profess to have given the work an attentive examination, but from the position of the author, and the favorable notices which it has received, together with the opinion of the translator just given, we have no doubt of its excellence. Its typograpical appearance is highly creditable to the publishers.

For sale by Derby & Co.

